# A Multicenter Retrospective Study of Epithelioid Trophoblastic Tumors to Identify the Outcomes, Prognostic Factors, and Therapeutic Strategies

**DOI:** 10.3389/fonc.2022.907045

**Published:** 2022-05-23

**Authors:** Wu Liu, Jianwei Zhou, Jie Yang, Xiufeng Huang

**Affiliations:** ^1^ Department of Obstetrics and Gynecology, Women’s Hospital, School of Medicine, Zhejiang University, Hangzhou, China; ^2^ Zhejiang Provincial Key Laboratory of Precision Diagnosis and Therapy for Major Gynecological Diseases, Women’s Hospital, Zhejiang University School of Medicine, Hangzhou, China; ^3^ Department of Gynecology, The Second Affiliated Hospital, School of Medicine, Zhejiang University, Hangzhou, China; ^4^ Department of Obstetrics and Gynecology, Sir Run Run Shaw Hospital, School of Medicine, Zhejiang University, Hangzhou, China

**Keywords:** epithelioid trophoblastic tumor, outcome, prognostic factor, therapeutic strategy, fertility preserving therapy, isolated pulmonary lesion

## Abstract

**Background:**

There is no consensus for the management of epithelioid trophoblastic tumor (ETT) up to date.

**Objective:**

ETT is the rarest form of gestational trophoblastic neplasia (GTN). Our goal was to assess the outcomes and explore the prognostic factors of patients with ETT through this multicenter retrospective analysis and to devise a risk-adapted approach to clinical management.

**Methods:**

A total of 31 patients were validated as ETT pathologically between January 2004 and June 2021 from three tertiary hospitals. We retrospectively analyzed the characteristics, treatments, outcomes, and prognostic factors.

**Results:**

Eight patients experienced a recurrence, and 6 patients died of ETT, resulting in a mortality rate of 19.4%. Five patients with stage I disease had a fertility-preserving treatment. Among them, one patient had a full-term delivery, whereas a 23-year-old patient who declined a hysterectomy died of a recurrent disease. Eight patients of extrauterine ETT with isolated pulmonary lesion were at a young age at diagnosis (median: 30.5 *vs*. 41, *p* = 0.003) and had a smaller tumor size (median: 2.4 *vs*. 4.8 cm, *p* = 0.003) compared with other patients who had a metastatic disease, and none of them died. The multivariate analyses showed that the number of metastases ≥3 [hazard ratio (HR), 28.16, *p* = 0.003] was the only significant predictor associated with adverse overall survival, while the number of metastases ≥3 (HR 9.59, *p* = 0.005) and chemotherapy alone (HR 16.42, *p* = 0.001) were associated with adverse recurrence-free survival. Patients in stage I or with number of metastases <3 had a favorable prognosis, whereas the prognosis of patients whose number of metastases ≥3 remains poor.

**Conclusions:**

Chemotherapy alone is insufficient for patients with ETT. Surgical procedures are the mainstay of management for ETT patients. Combined surgery and multi-agent chemotherapy are recommended for patients with metastatic disease and localized disease with persistently positive human chorionic gonadotrophin levels after surgery. The number of metastases at ≥3 is the most critical risk factor for ETT.

## Introduction

Gestational trophoblastic neoplasia (GTN) is a group of malignant diseases including invasive mole (IM), choriocarcinoma (CCA), placental site trophoblastic tumor (PSTT), and epithelioid trophoblastic tumor (ETT). ETT is the rarest form of GTN, accounting for less than 2.2% of all GTNs (1–3). Developing from the neoplastic transformation of chorionic-type intermediate trophoblast, ETT was initially described in 1998 by Shih and Kurman as a separate entity with features distinct from CCA and PSTT (4). This tumor was subsequently classified as a form of GTN by the World Health Organization (WHO) in 2003.

Previously, case reports in the literature revealed that patients with ETT often have clinical characteristics similar to those of PSTT, including slow growth rate, mildly to moderately elevated human chorionic gonadotrophin (hCG) level, and chemoresistance (2, 5–7). Both of them present predominantly at a reproductive age with abnormal vaginal bleeding, and the main type of antecedent pregnancy is term/preterm delivery. They are both relatively resistant to chemotherapy; therefore, the International Federation of Gynecology and Obstetrics (FIGO) risk scoring system is not associated with outcomes in PSTT or ETT. ETT usually presents in the lower uterine segment or cervix, resulting in a potential misdiagnosis of cervical squamous cell carcinoma, while PSTT is a typical tumor of the uterine corpus (4, 8–10). Pathologically, PSTT has a sheet-like and infiltrative growth pattern, while ETT has been shown to present with smaller, more monomorphic cells and a nested, well-circumscribed nodular growth pattern often with areas of necrosis (11). Immunohistochemically, PSTT shows strongly positive for human placental lactogen (hPL) and CD146 in a large proportion of tumor cells while focally positive for hCG in a small proportion of tumor cells (12). For ETT, hCG and hPL have a focal or patchy reactivity. Other markers, such as p63, cytokeratin, epidermal growth factor receptor, and E-cadherin, are present (4, 11, 13, 14). Sometimes misdiagnosed as choriocarcinoma, PSTT, or cervical squamous cell carcinoma, ETT has a unique treatment paradigm characterized by specific clinicopathological and immunohistochemical patterns.

Like PSTT, ETT is often resistant to chemotherapy, and surgery is the preferred treatment for ETT, with the disease confined to the uterus. However, extrauterine or metastatic disease occurs in 25–42% of the cases at the time of diagnosis, contributing to a mortality rate higher than 10% (4, 8, 9, 15). Few data are currently available on the prognostic factors of ETT. The prognostic factors of ETT are assumed to be similar to those described in patients with PSTT. Stage IV and an interval from antecedent pregnancy ≥48 months have been demonstrated to be associated with adverse prognosis in patients with PSTT (16, 17). Given its extreme rarity, our knowledge about ETT has been based generally on case reports or small series. Consequently, it is difficult to perform appropriate statistical analyses of risk factors. The extreme rarity of ETT impeded research about the prognostic factors for the prognosis.

Our colleagues have previously reported on the presentation and management of ETT, including an outcome-based literature review and case reports regarding mixed ETT and PSTT, fertility-preserving therapy, and extrauterine ETT (8, 14, 18–20). In this series, we aimed to assess the outcomes and explore the prognostic factors of patients with ETT through this multicenter retrospective analysis and devise a risk-adapted approach to clinical management.

## Materials and Methods

Patients were recruited from three hospitals: Women’s Hospital School of Medicine Zhejiang University, The Second Affiliated Hospital School of Medicine Zhejiang University, and Sir Run Shaw Hospital, School of Medicine Zhejiang University. Our three hospitals are tertiary referral and treatment institutions for GTN, and one of them is a GTN center in China. A total of 2,259 patients with GTN were treated at the three hospitals between January 2004 and June 2021. Among them, 34 patients were pathologically confirmed to have ETT by two or three subspecialty gynecologic pathologists from the three hospitals. Immunohistochemistry was used to confirm the diagnosis of ETT, except in two patients who underwent surgery at local hospitals without immunohistochemical staining results before their referral to our institutions. Patients would be excluded if the pathologists failed to reach an agreement on the pathological diagnosis of ETT due to the high rate of misdiagnosis. Besides this, one case of mixed ETT/choriocarcinoma and two cases of mixed ETT/PSTT were excluded. In total, 31 patients with ETT were analyzed in the present study.

The demographic, clinical, pathological, immunohistochemical, treatment, and outcome data of the patients were extracted. Follow-up information was obtained during reexamination at the three hospitals or through telephone conversations with the patients. This study complied with local regulations and was approved by the independent Clinical Research Ethics Committee of the three hospitals. The informed consent form was signed by all patients.

Clinical information, including age, type of antecedent pregnancy, interval since antecedent pregnancy, serum β-hCG level, FIGO stage, detectable lesions, maximum lesion diameters, surgical procedures, chemotherapy regimens, pathological results, and follow-up status, was recorded for each subject. Normal serum β-hCG levels were defined as less than 5 IU/L, and the serum β-hCG levels were monitored once per week during the treatment. All of the patients were staged by imaging exams, including pelvic ultrasound, chest computed tomography (CT), abdominal and brain CT, or magnetic resonance imaging if necessary. The serum β-hCG levels were monitored weekly for 1 month, then monthly for 3 months, every 6 months for 3 years, once a year until 5 years, and once every 2 years thereafter. This pattern and other follow-up examinations were conducted according to the national institutional standards.

Overall survival (OS) was defined as the time of first treatment to date of death or date last known alive by follow-up. Recurrence-free survival (RFS) was defined for patients with recurrent disease as the time of first treatment to date of recurrence or last known alive by follow-up. Complete remission/response (CR) was defined as cases in which the β-hCG level remained normal without evidence of disease progression within 3 months after treatment. Overall and recurrence-free survival estimates were calculated using the Kaplan–Meier method and compared using the log-rank test. Multivariate analysis was performed using the Cox proportional hazards regression model to identify the prognostic factors. The area under the curve (AUC) with 95% confidence interval (CI) was calculated using receiver operating characteristic (ROC) curve to assess the discriminatory potential of prognostic factors and to identify the optimal cutoff value of a continuous variable to differentiate between the probability of survival and death at 10 years. To evaluate between-group differences with respect to baseline characteristics, continuous data were assessed for a normal distribution by 1-sample Kolmogorov–Smirnov tests. Normally distributed data were compared by *t*-tests, while others were compared using nonparametric Wilcoxon–Mann–Whitney tests. Categorical data were assessed by Fisher’s exact tests (more than 20% of cells had expected frequencies of <5 in each factor). All statistical analyses were performed using the Statistical Package for the Social Sciences for Windows, version 21.0 (SPSS, Inc., Chicago, IL, USA). An alpha level <0.05 was considered statistically significant.

## Results

### The Demographic and Clinical Data of Patients

A total of 2,259 patients with GTN were registered at the three hospitals between January 2004 and June 2021. Among them, 31 patients were histologically validated as ETT with an incidence of 1.37% among all types of GTNs. The characteristics at baseline for all patients are summarized in **Table 1**.

**Table 1:** Characteristics of the patients at baseline.

Characteristics	Patients (*n* = 31)
**Age (year)**	**32 (range, 19–51)**
<40	25 (80.6%)
≥40	6 (19.4%)
**Antecedent pregnancy**	
Term or preterm	16 (51.6%)
Hydatidiform mole	4 (12.9%)
Abortion Ectopic pregnancy	10 (32.3%) 1 (3.2%)
**Interval since antecedent pregnancy (months)**	**49 (range, 2–268)**
<48 months	14 (45.2%)
≥48 months	17 (54.8%)
**Serum β-hCG level (IU/L)**	**630 (range, 0.4–174,315.5)**
<5 (normal)	1 (3.2%)
≥5 to <100	6 (19.4%)
≥100 to <1,000	11 (35.5%)
≥1,000 to <10,000	9 (29.0%)
≥10,000	4 (12.9%)
**Maximum lesion diameter (cm)**	**3.1 (range, 0.8–12)**
<2	6 (19.4%)
≥2 to <4	15 (48.4%)
≥4	10 (32.2%)
**Number of metastases**	
0	15 (48.4%)
1–2 ≥3	10 (32.2%) 6 (19.4%)
**FIGO stage**	
I	15 (48.4%)
II	2 (6.4%)
III	11 (35.5%)
IV	3 (9.7%)
**Treatment**	
Chemotherapy alone Surgery alone	4 (12.9%) 3 (9.7%)
Surgery and chemotherapy	24 (77.4%)

Continuous data were expressed as medians (range) and categorical data as absolute values (%).

β-hCG, beta human chorionic gonadotropin; FIGO, International Federation of Gynecology and Obstetrics. Bold values indicates continuous data.

A total of 16 patients in this series presented with abnormal vaginal bleeding; others presented with elevated serum β-hCG levels (*n* = 9), amenorrhea (*n* = 2), lung mass (*n* = 2), pelvic mass (*n* = 1), and hemoptysis (*n* = 1). The median age was 32 years (range, 19–51), and 80.6% (*n* = 25) of the patients were under the age of 40. The most common type of antecedent pregnancy was full-term or preterm delivery (*n* = 16), followed by abortion (*n* = 10), hydatidiform mole (*n* = 4), and ectopic pregnancy (*n* = 1). Moreover, 14 patients had an interval since antecedent pregnancy <48 months, and 17 patients had an interval ≥48 months, with a median interval since antecedent pregnancy to diagnosis of 49 months (range, 2–268). The maximum lesion diameter ranged from 0.8 to 12 cm, with a median diameter of 3.1 cm. The primary tumor site included the uterine corpus (*n* = 14), the lower uterine segment/cervix (*n* = 9), and lung without uterine involvement (*n* = 8).

Unlike the other forms of GTN with a highly elevated serum β-hCG level at diagnosis, 18 of 31 patients had a slightly elevated or normal β-hCG level (<1,000 IU/L) in our series, with a median β-hCG of 630. According to the FIGO clinical stage system, 15 patients were in stage I, 2 were in stage II, 11 were in stage III, and 3 were in stage IV (**Table 2**). Among the 16 patients with metastatic disease, 9 patients had a solitary metastasis, including 8 patients with an isolated lesion of the lung without uterine involvement, 1 patient who had two metastases, and 6 patients who had three or more metastases.

**Table 2:** Treatments and outcomes of the patients.

All patients with ETT	All*n* = 31	Death*n* = 6	Recurrence*n* = 8
FIGO stage I	*n* = 15		
Surgery and chemotherapy	10		
Chemotherapy without surgery	2	1	2
Surgery without chemotherapy	3		
FIGO stage II	*n* = 2		
Surgery and chemotherapy	2		1
FIGO stage III	*n* = 11		
Surgery and chemotherapy	9	2	2
Chemotherapy without surgery	2	1	2
FIGO stage IV	*n* = 3		
Surgery and chemotherapy	3	2	1

FIGO, International Federation of Gynecology and Obstetrics.

### Treatment and Outcome

**Figure 1:**
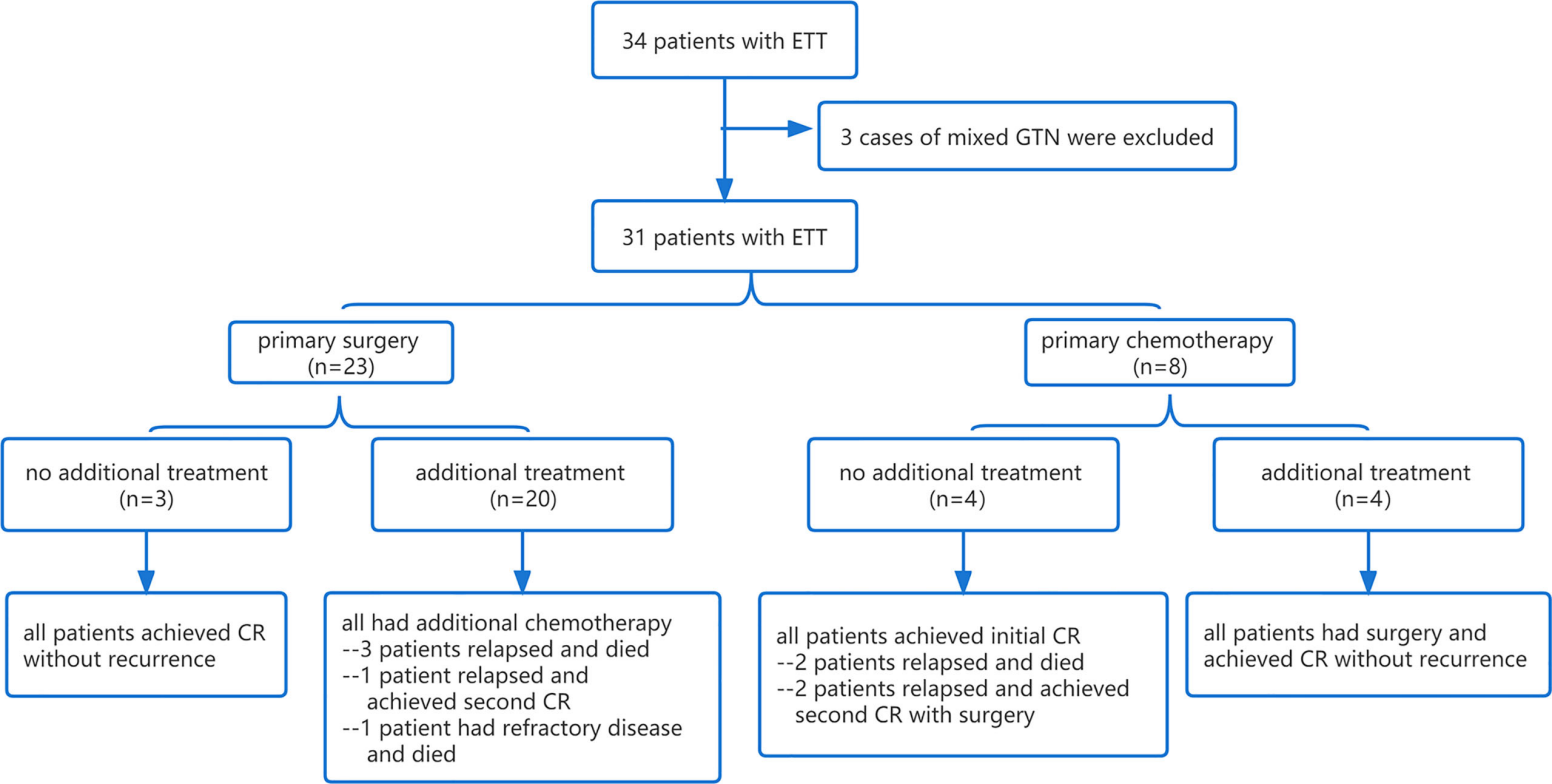
Flowchart of the treatment and response of 31 patients with ETT. ETT, epithelioid trophoblastic tumor; GTN, gestational trophoblastic neoplasia; CR, complete response.

Due to the low chemotherapy response rates in comparison to choriocarcinoma and invasive mole, surgical procedures play a critical role in treating ETT. The treatments and outcomes are shown in detail in **Table 2** and **Figure 1**.

Only three patients underwent primary hysterectomy without adjuvant chemotherapy, and they all achieved long-term survival without recurrence or death at the last follow-up. Of the three patients, one presented with a normal serum β-hCG level and a 12-cm mass in the uterine corpus, which was initially misdiagnosed as a giant uterine fibroid before the pathological diagnosis. The other two were misdiagnosed as cesarean scar pregnancy or cornual pregnancy since the tumor sites were in the lower uterine segment and the uterine horn, with slightly elevated (7,13I U/L) and moderately elevated (8,668 IU/L) β-hCG levels, respectively. Both of them achieved rapid normalization of β-hCG level after hysterectomy.

The main chemotherapy regimens in the present study were EP/EMA (etoposide, cisplatin, etoposide, methotrexate, and actinomycin-D) and EMA/CO (etoposide, methotrexate, actinomycin-D, cyclophosphamide, and vincristine). Four patients achieved an initial complete remission by multi-agent chemotherapy without additional surgery. Two of them relapsed at 6 and 23 months of follow-up and achieved second CR eventually after surgery (1 case underwent hysterectomy and 1 case underwent hysterectomy and excision of an isolated pulmonary lesion) combined with adjuvant chemotherapy, while the other 2 patients relapsed at 8 and 16 months after treatment and died of recurrent disease.

Twenty patients were treated with initial surgery. The main surgical approach was hysterectomy (*n* = 11), followed by excision of an isolated pulmonary lesion (*n* = 6) and excision of a uterine lesion (*n* = 3). Nine patients who achieved normal β-hCG levels after surgery still had postoperative chemotherapy in case of recurrence. The remaining 11 patients had additional chemotherapy with abnormal β-hCG levels after surgery; however, it is unclear whether they would achieve normal β-hCG levels by surgery alone since the intervals between surgery and postoperative chemotherapy were too short. In this subgroup, one patient achieved second CR after recurrence, one patient died of refractory disease with multiple metastases in the lung and brain and who failed to achieve initial CR, and three patients died of recurrent disease.

Four patients who had chemotherapy as initial treatment achieved only an incomplete response; thus, additional surgical procedures were performed, including hysterectomy in 2 cases, excision of uterine lesion in 1 case, and resection of isolated pulmonary lesion in 1 case. All of the four patients achieved CR without recurrence.

**Figure 2:**
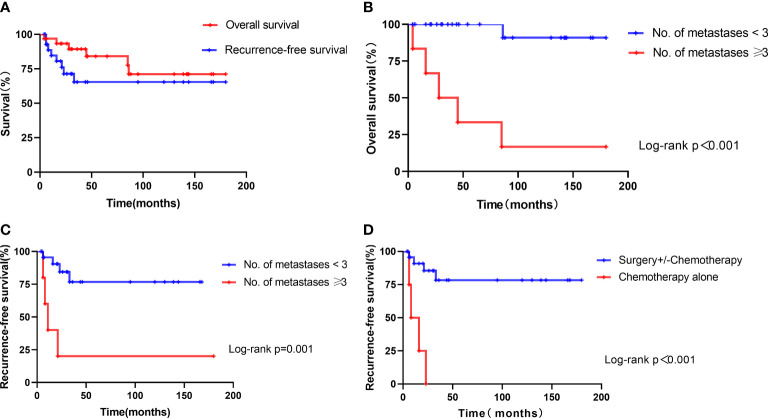
Kaplan–Meier estimate of survival in 31 patients with ETT. **(A)** Overall survival and recurrence-free survival of all patients. **(B)** Overall survival of patients with the number of metastases ≥3 *versus* those with the number of metastases <3. **(C)** Recurrence-free survival of patients with the number of metastases ≥3 *versus* those with the number of metastases <3. **(D)** Recurrence-free survival of patients treated with chemotherapy alone *versus* those with surgery+/-chemotherapy.

Overall, after 4 to 180 months of follow-up (median: 46 months), 8 patients experienced a recurrence and 6 patients died of ETT, resulting in a mortality rate of 19.4%. The probabilities of OS for patients in this series were 84.0% at 5 years and 71.1% at 10 years, and the probability of recurrence-free survival was 65.3% at both 5 and 10 years (**Figure 2A**).

### Fertility-Preserving Therapy

Four patients with disease confined to the uterus had fertility-preserving surgery, including abdominal resection of lesion in the uterus (*n* = 2) and resection of lesion by hysteroscopy (*n* = 2). One 23-year-old patient in stage I who declined hysterectomy to preserve her fertility was treated with chemotherapy alone; however, she experienced a recurrence and died of progressive disease thereafter. All of the 5 patients had multi-agent chemotherapy either as initial treatment or after surgery. Among them, one patient delivered at term *via* cesarean section. Another patient who has been reported previously (20) experienced a first-trimester miscarriage later.

### Isolated Pulmonary Lesion

In the present study, a total of 8 patients in stage III presented with isolated pulmonary lesion without uterine involvement. Among them, 6 underwent excision of the isolated pulmonary lesion as initial treatment without hysterectomy. The remaining two suspected to be CCA or IM had chemotherapy as initial treatment. However, one experienced a recurrence in 6 months and subsequently achieved a second CR with hysterectomy (the hysterectomy specimen was histologically normal) and excision of the isolated lesion of the lung. The other one achieved initial CR with additional excision of the isolated pulmonary lesion. Patients with isolated pulmonary lesion had a better outcome; no one died of ETT, although their cases were classified as stage III. However, they presented at a younger age at diagnosis (median: 30.5 *vs*. 41, *p* = 0.003), with a smaller tumor size (median: 2.4 *vs*. 4.8 cm, *p* = 0.003) compared with other patients who had a metastatic disease (**Table 3**). Shorter intervals since antecedent pregnancy and lower β-hCG levels were also observed in this group, but not significantly.

**Table 3:** Comparison of the clinical data between the two groups of patients with metastatic diseases.

Characteristics	Isolated pulmonary lesion (*n* = 8)	Other metastatic diseases (*n* = 8)	*p*-value
**Age (year)**	30.5 (23–37)	41 (31–51)	**0.003**
**AP**			0.619
Term or preterm	5 (62.5)	3 (37.5)	
Other	3 (37.5)	5 (62.5)	
**ISAP (months)**	18 (3–77)	77 (8–268)	0.064
**β-hCG (IU/L)**	213 (30.5–2,247.7)	1,038.5 (22.5–39,600)	0.146
**Maximum lesion diameter (cm)** ^a^	2.4 (0.9–3.2)	4.8 (3.0–10.4)	**0.003**

Continuous data were expressed as median (range) and categorical data as absolute values (%).

a The maximal diameter refer to the largest lesions, including both metastases and the primary tumor.

AP, antecedent pregnancy; ISAP, interval since antecedent pregnancy; β-hCG, beta human chorionic gonadotropin. Bold values indicates a p-value <0.05.

### Prognostic Factors

The univariate analyses showed that the number of metastases ≥3 and FIGO stages II–IV were significant predictors of overall survival. The number of metastases ≥3, FIGO stages II–IV, and chemotherapy alone (surgery +/- chemotherapy *vs*. chemotherapy alone) were significant predictors of recurrence-free survival. Other variables, such as age, type of antecedent pregnancy, interval since antecedent pregnancy, pre-treatment β-hCG, and tumor size, were not significant predictors for OS or RFS of patients with ETT (*p* > 0.05). However, multivariate analyses showed that the number of metastases ≥3 (HR 28.16, *p* = 0.003) was the only a significant predictor associated with adverse OS, while the number of metastases ≥3 (HR 9.59, *p* = 0.005) and chemotherapy alone (HR 16.42, *p* = 0.001) were associated with adverse RFS (**Table 4**).

**Table 4:** Univariate and multivariate analyses of the prognostic factors associated with 5-year overall and recurrence-free survival.

Factors	Overall survival			Recurrence-free survival		
	Univariate analysis		Multivariate analysis	Univariate analysis		Multivariate analysis
	Probability (%)	*P*-value	*P*-value	Probability (%)	*P*-value	*P*-value
**Age (years)**		0.599	0.071		0.803	0.864
<40	86.5			69.9		
≥40	80.0			50.0		
**AP**		0.882	0.594		0.950	0.620
Term or preterm	85.2			68.8		
Mole or abortion	83.1			63.3		
**ISAP (month)**		0.596	0.535		0.149	0.432
<48	92.9			83.9		
≥48	77.6			49.1		
**β-hCG (IU/L)**		0.202	0.217		0.455	0.681
<1,000	80.1			63.8		
≥1,000	90.9			71.6		
**Maximum lesion diameter**		0.445	0.492		0.739	0.611
<4 cm	95.0			71.2		
≥4 cm	65.5			57.1		
**Number of metastases**		**<0.001**	**0.003**		**0.001**	**0.005**
<3	100			76.7		
≥3	33.3			20.0		
**FIGO stage**		**0.032**	0.530		**0.035**	0.063
I	88.9			83.1		
II–IV	67.8			38.1		
**Treatment**		0.285	0.538		**<0.001**	**0.001**
Surgery+/-chemotherapy	81.2			78.4		
Chemotherapy alone	100			0.00		

AP, antecedent pregnancy; ISAP, interval since antecedent pregnancy; hCG, human chorionic gonadotropin; FIGO, International Federation of Gynecology and Obstetrics. Bold values indicates a p-value <0.05.

Thus, the number of metastases was associated with OS significantly, and the ROC analysis showed an AUC of 0.86 (95% CI, 0.638–1.082). The optimal cutoff point of the number of metastases of 3 could differentiate the probability of OS or death, with 83.3% sensitivity and 96.0% specificity for the highest Youden’s index. Of the 6 patients who died, 5 patients had the number of metastases ≥3, whereas the remaining one had the number of metastases <3 (HR 26.26, 95% CI 2.96–233.0, *p* **<** 0.001) (**Figure 2B**).

The Kaplan–Meier analyses indicated that the mean overall survival time was 171.5 months in patients with the number of metastases <3 (95% CI: 155.5–187.4) and 59.7 months in patients with the number of metastases ≥3 (95% CI: 11.9–107.4). The overall survival of patients with the number of metastases <3 was better than those with the number of metastases ≥3 (*p* < 0.001) (**Figure 2B**). The mean recurrence-free survival time was 133.9 months in patients with the number of metastases <3 (95% CI: 103.9–163.9), 45.2 months in patients with the number of metastases ≥3 (95% CI: 0–104.5), 13.3 months in patients treated with chemotherapy alone (95% CI: 5.6–20.9), and 145.4 months in patients treated with surgery+/-chemotherapy (95% CI: 114.7–176.0). The recurrence-free survival of patients with the number of metastases ≥3 or treated with chemotherapy alone was worse than those of others (**Figures 2C** **, D**).

## Discussion

Data regarding Asian populations is essential since the incidence of GTNs in Asian women is much higher compared with those in non-Asian women (21, 22). However, the data of the largest series of ETT predominantly originated from western countries without Asian medical centers (23). To our best knowledge, the present study was the largest series in Asian women to evaluate the outcomes and prognostic factors of ETT to date, with 31 cases recruited from 3 referral centers.

The incidence of ETT in the present study was 1.37% among all types of GTNs, which is concordant with the reports in the literature (1–3). Similar to previous studies (2, 9, 23, 24), term or preterm delivery was the most common type of antecedent pregnancy, and the majority of patients presented with abnormal vaginal bleeding at a reproductive age, with a median age of 32 years. The serum β-hCG levels are moderately increased in patients with ETT, while CCA and IM usually present with highly elevated serum β-hCG levels (>10,000 IU/L) (2, 8, 23, 24). A total of 22 (71.0%) patients had β-hCG levels <2,500 IU/L in this series. In contrast to PSTT, which is a typical tumor of the uterine corpus, ETT usually presents in the lower uterine segment or cervix, resulting in a potential misdiagnosis of carcinoma of the uterine cervix, and 25–42% of the ETT patients presented with metastatic or extra-uterine disease at the time of presentation (2, 9, 15, 23). In the present study, 16 (51.6%) patients had metastatic disease at diagnosis, including 8 patients with isolated pulmonary lesion without uterine involvement. Prior to the pathological diagnosis, our patients were also initially misdiagnosed as cases of ectopic pregnancy, caesarean scar pregnancy, uterine fibroid, cornual pregnancy, carcinoma of the uterine cervix, or other forms of GTN due to the unique tumor sites.

Chemotherapy alone is insufficient for patients with ETT either in the early stage or in the advanced stage since ETT is relatively chemoresistant. In the series of Frijstein et al. (23), 45 patients with ETT and 9 patients with PSTT/ETT from the ISSTD database showed that patients who underwent chemotherapy with or without surgery had a less favorable outcome as 11 of them did not survive. Yang et al. (2) reported that 2 patients treated with chemotherapy alone failed to achieve complete remission prior to referral to their hospital, and one of them died unfortunately. Consistent with previous studies, 8 patients had chemotherapy alone as initial treatment in the present series. Among them, 4 patients achieved only a partial response, and additional surgical procedures were performed to achieve initial CR. Four other patients who achieved initial complete remission by multi-agent chemotherapy without additional surgery all experienced a recurrence. Moreover, one case in stage I and another one in stage III died of recurrent disease. The probabilities of OS for patients treated with chemotherapy alone were 100.0% at 5 years and 33.3%% at 10 years, and the probability of RFS was 0.0% at both 5 and 10 years. Nevertheless, adjuvant chemotherapy still plays a crucial role in the management of ETT. In the literature (6, 23, 25), platinum/etoposide-containing regimens are advocated for patients with ETT, especially for patients with persistently positive β-hCG levels after surgery and with metastatic disease or when surgery is unfeasible. The majority of the chemotherapy regimens in this series were EP/EMA and EMA/CO.

Surgery is the mainstay of treatment for ETT, which is attributed to the low response rate to chemotherapy. Studies indicated that hysterectomy is the primary treatment when the disease is confined to the uterus, whereas a multimodality approach is needed for advanced disease (2, 9, 11, 23, 26). For patients in stage I in our series, 3 patients underwent hysterectomy without adjuvant chemotherapy and 10 patients underwent surgery (6 hysterectomy and 4 excision of uterine lesion) combined with chemotherapy, and they all achieved long-term survival without recurrence or death at last follow-up. For patients in stages II–IV, 14 underwent combined surgery and chemotherapy (**Table 2**). Among them, 2 patients in stage III and 2 patients in stage IV died. Controversy exists regarding whether patients with stage I disease benefit from adjuvant chemotherapy. Surgery alone seems to be sufficient for patients with normal serum β-hCG level in stage I (8, 9); however, it is still uncertain. In our series, only 3 patients underwent hysterectomy alone, and the other patients with normal β-hCG levels postoperatively still had adjuvant chemotherapy in case of recurrence. Surgical procedures, including resection of metastases, combined with multi-agent adjuvant chemotherapy are recommended for advanced-stage disease.

### Fertility-Preserving Therapy

The consideration of fertility-preserving therapy is critical since most patients with ETT are in the reproductive age. Few data are currently available on fertility-preserving therapy of patients with ETT. Tse et al. (27) described one successful case of a 25-year-old woman who underwent an emergency laparotomy for an acute abdominal pain. A posterior uterine cystic lesion was excised without hysterectomy or adjuvant chemotherapy and was histologically validated as ETT. She had a full-term delivery by cesarean section and another full-term normal vaginal delivery at about 4 and 7.5 years after her initial diagnosis, respectively. Another successful case of a 32-year-old woman was reported by Imamura et al. (28) but validated as ETT mixed with choriocarcinoma histologically. Conversely, Davis et al. (9) reported on 2 ETT cases of treatment failure. One had disease progression with metastasis to the lung, and the other one had a subsequent spontaneous abortion followed by cervical recurrence of ETT. In our series, 5 patients with stage I disease had a fertility-preserving treatment. One patient had a full-term delivery by cesarean section, whereas a 23-year-old patient died of recurrent disease with multiple metastases to the lung. The delay in instigating hysterectomy may account for the recurrence and death. Nonetheless, fertility-preserving surgery would possibly be a feasible strategy for the patients with localized disease and whose lesions can be completely removed. Given these discrepant results, the role of fertility-preserving therapy is still controversial and requires careful counseling about the potential risk of recurrence or death.

### Isolated Pulmonary Lesion

ETT is considered to be an organ-confined disease, and the uterus is the most common primary site, while the lung is the most common extra-uterine site of metastasis (2, 23, 29). Approximately 10 cases of extrauterine ETT of the lung without uterine involvement have been reported in the literature (2, 23, 30–33). In the present study, 8 patients of ETT presented with an isolated pulmonary lesion. Among them, one has also been reported separately (19). A high proportion (25.8%) of this group may be attributed to the high referral rate of patients with isolated pulmonary lesion treated from the south of China. Excision of the isolated pulmonary lesion without hysterectomy was performed in 7 cases. The remaining one recurrent case who had chemotherapy alone as initial treatment underwent hysterectomy (the finding of which was benign) and excision of an isolated lesion of the lung to achieve second CR. The majority of these patients achieved normal β-hCG level after surgery but still had postoperative chemotherapy in case of recurrence. None of them died, resulting in a favorable short-term clinical outcome (median: 30.5 months, range: 4–54 months). Hysterectomy seems to be unnecessary, but such cases will require a longer follow-up. Our results are concordant with the findings in the literature. There is currently insufficient data to explain the existence of ETT apparently limited to the lung. The original transformation of trophoblastic cells passed to the lung during antecedent pregnancy and the spontaneous resolution of an antecedent uterine ETT may account for this unique clinical phenomenon according to other researchers (30). An interesting observation is that the patients in our series presented at a younger age with a smaller tumor size, a shorter interval since antecedent pregnancy, and a lower β-hCG level which has not been reported previously in the literature. In view of this, spontaneous resolution may be more critical. Given the limited data, the studies failed to make a uniform consensus on the necessity of postoperative chemotherapy, but excision of the isolated pulmonary lesion without hysterectomy is feasible among these patients who want to preserve their fertility.

### Prognostic Factors

Exploration of prognostic factors is limited by the extreme rarity of ETT and the difficulties of performing statistical analyses in studies with a small sample size. Similar to PSTT, FIGO stage is a significant prognostic factor for ETT. A literature review of 78 cases reported by Zhang et al. (8) indicated that FIGO stages II–IV were the only significant prognostic indicator for ETT. Stage IV disease was the only significant predictor of overall and disease-free survival on both univariate and multivariate analyses in the series of Yang et al. (2). A total of 54 patients of ETT from the ISSTD database described by Frijstein et al. revealed that stages II–IV disease and an interval of ≥48 months since the antecedent pregnancy were prognostic factors of overall survival (23). Other factors like age, type of antecedent pregnancy, tumor size, and serum β-hCG levels do not seem to be associated with the clinical outcome. In our series, all the patients who achieved CR by chemotherapy alone relapsed. Furthermore, 5 of 6 patients who had three or more metastases died, resulting in probabilities of OS of 33.3% at 5 years and 16.7% at 10 years, whereas the probabilities of OS at 5 and 10 years were 100 and 90.9% in the group of patients with metastases <3. We demonstrated that the number of metastases ≥3 and FIGO stages II–IV were significant predictors of overall survival, and the number of metastases ≥3, FIGO stages II–IV and chemotherapy alone (surgery +/- chemotherapy *vs*. chemotherapy alone) were significant predictors of recurrence-free survival on univariate analysis. However, the multivariate analyses showed that the number of metastases ≥3 (HR 28.16, *p* = 0.003) was the only significant independent predictor of adverse OS, while the number of metastases ≥3 (HR 9.59, *p* = 0.005) and chemotherapy alone (HR 16.42, *p* = 0.001) were significant independent predictors of adverse RFS.

It is widely accepted that advanced stage was a risk factor for poor prognosis; however, it was not significant for OS and RFS on multivariate analysis in our series (*p* = 0.530 and *p* = 0.063, respectively), although stages II–IV were associated with poor survival on univariate analysis (*p* = 0.032). This could be partly explained by the favorable outcomes of patients in stage III with isolated pulmonary lesion and the poor outcomes of patients with multiple metastases to the lung or brain when the metastatic lesions cannot be removed completely. Considering this, the number of metastases ≥3 may be a more useful and accurate predictor for poor prognosis. The optimum discriminator for survival was 3 metastases, which might be attributed mainly to the failure of complete resection of all metastatic lesions when the number of metastases ≥3.

Although this is the largest case series of ETT patients in Asian women with key novel findings, it should be noted that it is still a relatively small number of patients. Some potential risk factors (such as deep myometrial involvement, vascular involvement, mitotic rate, and molecular markers) might not be fully evaluated due to the retrospective nature of the present study, and hysterectomy was not performed in more than 10 patients.

## Conclusions

Rather than replicating previous findings, we sought new discoveries instead through this retrospective study. We found that chemotherapy alone is insufficient for patients with ETT either in the early stage or in the advanced stage since ETT is relatively chemoresistant. Surgical procedures, including hysterectomy and resection of metastatic lesions, are the mainstay of management for ETT patients. Combined surgery and multi-agent chemotherapy are recommended for patients with metastatic disease and localized disease with persistently positive hCG levels after surgery. Excision of the isolated pulmonary lesion without hysterectomy is acceptable. However, the role of postoperative chemotherapy for patients in stage I or with isolated pulmonary lesion is controversial when they achieve normal β-hCG levels by surgery alone. The number of metastases ≥3 was the only significant predictor associated with OS, while the number of metastases ≥3 and chemotherapy alone were the significant independent predictors of RFS. Fertility-preserving treatment should be taken into consideration only in highly selected patients who strongly desire to preserve their fertility. To date, the prognosis of patients with the number of metastases ≥3 remains poor, although the majority of these patients received combined surgery and chemotherapy. Further studies on the exploration of potentially valuable therapeutic strategies, including immunotherapy and targeted therapies for these patients, are urgently needed.

## Data Availability Statement

The raw data supporting the conclusions of this article will be made available by the authors without undue reservation.

## Author Contributions

WL: project development, data curation, formal analysis, software, and writing—original draft. JZ: data curation, methodology, and writing—original draft. JY: data curation, methodology, and writing—original draft. XH: project development, supervision, and writing—review and editing. All authors contributed to the article and approved the submitted version.

## Conflict of Interest

The authors declare that the research was conducted in the absence of any commercial or financial relationships that could be construed as a potential conflict of interest.

## Publisher’s Note

All claims expressed in this article are solely those of the authors and do not necessarily represent those of their affiliated organizations, or those of the publisher, the editors and the reviewers. Any product that may be evaluated in this article, or claim that may be made by its manufacturer, is not guaranteed or endorsed by the publisher.
